# Biodegradable Magnesium Alloys for Personalised Temporary Implants

**DOI:** 10.3390/jfb14080400

**Published:** 2023-07-27

**Authors:** Radu Emil Hendea, Doina Raducanu, Adrián Claver, José Antonio García, Vasile Danut Cojocaru, Anna Nocivin, Doina Stanciu, Nicolae Serban, Steliana Ivanescu, Corneliu Trisca-Rusu, Radu Septimiu Campian

**Affiliations:** 1 Department of Oral Rehabilitation, Faculty of Dental Medicine, Iuliu Hatieganu University of Medicine and Pharmacy, 400349 Cluj-Napoca, Romania; radu.hendea@umfcluj.ro (R.E.H.); rcampian@umfcluj.ro (R.S.C.); 2 Department of Metallic Materials Processing and Ecometallurgy, University Politehnica of Bucharest, 060042 Bucharest, Romania; dan.cojocaru@upb.ro (V.D.C.); nicolae.serban@upb.ro (N.S.); 3 Institute for Advanced Materials and Mathematics (INAMAT2), Universidad Pública de Navarra (UPNA), 31006 Pamplona, Spain; adrian.claver@unavarra.es (A.C.); joseantonio.garcia@unavarra.es (J.A.G.); 4 Faculty of Mechanical, Industrial and Maritime Engineering, OVIDIUS University of Constanta, 900527 Constanța, Romania; anocivin@univ-ovidius.ro; 5 Zircon Dent SRL, 400690 Cluj-Napoca, Romania; doinastanciu@zircondent.ro (D.S.); stelianaivanescu@zircondent.ro (S.I.); 6 National Institute for Research and Development in Micro-Technologies, 077190 Bucharest, Romania; corneliu.trisca@imt.ro

**Keywords:** temporary personalised implants, biodegradable magnesium alloy, laser powder bed fusion 3D additive manufacturing, microstructural analysis, mechanical analysis, corrosion analysis

## Abstract

The objective of this experimental work was to examine and characterise the route for obtaining demonstrative temporary biodegradable personalised implants from the Mg alloy Mg-10Zn-0.5Zr-0.8Ca (wt.%). This studied Mg alloy was obtained in its powder state using the mechanical alloying method, with shape and size characteristics suitable for ensuing 3D additive manufacturing using the SLM (selective laser melting) procedure. The SLM procedure was applied to various processing parameters. All obtained samples were characterised microstructurally (using XRD—X-ray diffraction, and SEM—scanning electron microscopy); mechanically, by applying a compression test; and, finally, from a corrosion resistance viewpoint. Using the optimal test processing parameters, a few demonstrative temporary implants of small dimensions were made via the SLM method. Our conclusion is that mechanical alloying combined with SLM processing has good potential to manage 3D additive manufacturing for personalised temporary biodegradable implants of magnesium alloys. The compression tests show results closer to those of human bones compared to other potential metallic alloys. The applied corrosion test shows result comparable with that of the commercial magnesium alloy ZK60.

## 1. Introduction

Prolonged human lifespan and increases in elderly populations with inherent musculoskeletal diseases, as well as the increased severity of life, work, or sports accidents, cause bone damage in millions of people, requiring nearly 2.8 million bone repair surgeries annually worldwide, most of which use permanent or temporary implants [1]. More than 3.5 million accidents occur annually in China, the most populous country, with 70% of injured people requiring bone repair interventions [2,3]. 

Standard implants, with various ranges in sizes, are designed to fit different groups of patients since it is impossible to perfectly match the anatomy of each individual [4,5,6]. Therefore, the surgeon must size and/or shape either the standard implant or the patient’s bone to ensure a precise fit [4,5,6], a procedure that involves time-consuming manual processes, additional labour, and cost [6,7]. In recent years, the trend of personalisation in medicine has been present not only in orthopaedics but also in maxillofacial surgery, with treatments adapted to the specific requirements of the patient based on the designing and manufacturing of personalised implants [7,8].

Three-dimensional printing via additive manufacturing is now recognised as the most appropriate method for producing anatomically adjusted customised implants [7,9,10,11,12]. MRI (Magnetic Resonance Imaging) and CT (Computer Tomography) scans of the patient’s bones provide precise measurement, helping the 3D-printed implants to fit and be more efficient for the surgical procedure [13]. The market for personalised 3D-printed implants manufactured from different biomaterials is expected to show impressive growth between 2020 and 2028 and grow from USD 47.36 to 69.28 billion [13]. 

Currently, the different commercial implants are made of metallic materials such as stainless steel, Co-Cr, Ti and Ti alloys, which have good mechanical properties (mechanical strength and fracture resistance) [14,15,16,17]. But, for many years, in vitro and in vivo investigations and medical experience have shown that metal particles or ions from these implants, and less frequently from Ti-based implants, can be released via abrasion and corrosion, triggering an inflammatory response and leading to tissue loss, ultimately necessitating additional painful and costly surgery for implant removal [18,19]. Moreover, the mechanical properties of these metal implants are higher compared to those of bone [3], as shown in Table 1. 

Due to the difference in properties between bone and mechanical implants, the effect of stress shielding arises. This is caused by shear stress, subsequently inducing osteoporosis, osteolysis and even secondary fractures [15,20,21]. Consequently, biodegradable implants are necessary to avoid subsequent surgery, which carries the risk of complications, trauma for the patients and their families, and increased healthcare costs. That is why, in the last two decades, much effort has been made in the development of highly biocompatible biodegradable materials [15,22].

The biodegradable implant biomaterials must fulfil two main conditions: (1) harmless dissolution until the damaged bone is completely healed, with high biocompatibility of the dissolved products; (2) slow decrease in mechanical strength/stiffness, while the surrounding tissue regains strength/stiffness, thus sustaining the bone healing and gradually transferring the load from the implant to the bone tissue [1,2,3]. 

For the treatment of damaged bones with temporary implants, the advantages of biodegradable Mg-based alloys, compared to other biodegradable materials such as natural and synthetic polymers, are their higher mechanical strength and the total biocompatibility of the ions released by the implant’s degradation in the human body [14,21,22,23]. Mg-based implants also have another important advantage, radiopacity, being easily visible on radiographs, CT and MRI for the precise investigations required for patient diagnosis and treatment planning [24]. This implant material also has the ability to resist sterilisation using the various methods usually applied, which are required before implantation to prevent possible infections [24]. All these favourable characteristics of Mg alloys can greatly contribute to the success of implantation [24].

Biodegradable Mg-based implants have great potential for use not only in orthopaedics but also in the oro-maxillofacial surgical treatment of injuries and defects of various parts of the face, including the jaw and neck [24]. The main challenge still facing Mg-based biodegradable materials is correlated to their mechanical characteristics and harmless dissolution rate in the human body with the rate of bone restoration [1,2,3]. Therefore, for achieving the alloy’s suitable mechanical and corrosion properties and a good production cost, the selection of the alloy composition with highly biocompatible elements and also of the appropriate technology for implant manufacturing is of maximum importance. At the same time, the controlled internal architecture/porosity of the implant can improve not only its osteo-conductivity via the good circulation of biological fluids but also its mechanical properties [22,25,26]. 

Recently, ZK (Mg, Zn, and Zr) alloys have attracted the attention of researchers due to the better biocompatibility of the constituent elements than that of AZ (Mg, Al, and Zn) and WE (Mg, Zn, and RE) alloys [27,28]. Aluminium ions are known for the neurotoxicity and brain disorders (Alzheimer’s) they induce, and elements such as Y, Ce, and Pr cause severe hepatotoxicity, even if in small amounts, and they can be tolerated and accepted due to the beneficial effect of reducing the corrosion rate of the alloy [28]. For ZK alloys, the use of a varied content of biocompatible alloying elements (such as Ca, Zn, Zr, Mn, Si, and Ag) is of great interest in research due to their biosafety [29,30]. Calcium is the main mineral component of human bone. Its amount is regulated by skeletal, renal, and intestinal homeostasis [14]. Zinc is an essential micronutrient involved in the regulation of the immune system and enzymatic reactions [14,23]. Its antimicrobial action avoids bacterial infection at the implantation site [24]. Zinc presence in magnesium alloys improves mechanical characteristics, slows down the degradation rate, and increases the proliferation of osteoblastic cells for bone rebuilding [23]. Degraded zinc is eliminated from the body through the gastrointestinal tract, urine, and skin [13,31]. Zirconium has good biocompatibility and low ionic toxicity and, added in small amounts to the composition of the alloy, improves both corrosion and mechanical characteristics [13]. Zr accumulates in the bone and nervous system, but in small amounts, it is not dangerous, being bioinert [14]. 

After many investigation efforts and progress in this field, some small-sized biodegradable commercial implants such as magnesium alloys screws, pins, suture anchors and plates are available in the market, produced by the companies Syntellix AG (Hannover, Germany) using MAGNEZIX®/Mg-Y-RE-Zr alloy, Aap Implantate AG (Berlin, Germany), MeKo Laser Material Processing eK (Sarstedt-Hannover, Germany) using RESOLLOY, Medical magnesium GmbH (Aachen, Germany), Synthes GmbH (Oberdorf, Switzerland) using Mg–Y–RE–Zr alloy, HCM Orthocare (Ahmedabad, Gujarat, India) using MagOrtho alloy, MAGNEZIT GROUP Europe GmbH (Ratingen, Germany), and U&I Corp. (Gyeonggi, South Korea) using RESOMET™/Mg-Ca alloy [14,24]. The German producers, Aap Implantate AG and MeKo Laser Material Processing, supplied not only standard biodegradable Mg-based implants but also customised ones [24]. Now, Mg bio-alloys are still considered only suitable for small and low-load implants [4,14] because of their fast degradation rate that is not yet sufficiently correlated with the bone healing process [32,33,34,35]. 

Another important aspect that should be considered is the implant manufacturing technique that directly affects the structure of metallic materials, their surface and subsurface properties, and the bio-functionality and fatigue resistance of biomedical implants [36]. Until recently, this manufacturing process has been investigated and applied commercially to obtain small Mg-based implants (pins, clips, and screws) via bulk alloy processing and extrusion of high-strength Mg alloys from powders. Starting in 2009, biodegradable implants began to be obtained using the method of powder metallurgy and additive manufacturing (AM), using Selective Laser Melting (SLM), which allows for full design freedom [37,38,39]. In addition, this technology can produce controlled porosities capable of promoting bone regeneration. At the same time, the possibility of quick preparation of a 3D-printed model allows the surgeon’s communication with the patient so that the patient obtains an intuitive understanding of its illness and treatment [40]. Currently, a laser-based additive manufacturing process is applied for commercial alloys AZ61, WE43, and ZK60, even if their biological reactions in vivo have not been verified systematically yet [2,41,42,43,44].

In brief, the AM-SLM method is a powder bed fusion process using a high-density laser beam (L-PBF) on a micro scale, which allows for the rapid manufacture of metal parts with the desired geometry without the need for post-processing or possibly only a very small one [45]. The pre-processing step of L-PBF consists of the CAD design of the model based on doctors’ digital information for the customised implant or based on the shape and dimensions of standard implants. The design is converted to a stereo-lithography (STL) file by slicing the 3D model into thin layers. Then, it is necessary to design and set the process parameters corresponding to the specific characteristics of the metal powder to be processed and the desired geometry. The next step is 3D printing using the SLM machine software (CAMbridge 2018, 3Shape A/S, Copenhagen, Denmark), which takes the slicing data from the STL, scans each layer of powder fed successively into the building chamber until the designed geometry is achieved [45]. The quality of the manufactured product depends on the selection of the right process parameters, such as laser power, scan speed, layers, and hatch distance, including their correlation [45]. For reactive metals, such as Mg and its alloy, the 3D printing must occur in an inert Ar atmosphere. Each new material needs extensive experiments to establish the optimum range of parameters for 3D printing from powder feedstock, with adjustments for the specific structural porosity and shape.

This present paper concerns the attempt to obtain a demonstrative temporary implant from a Mg bio-alloy with the composition Mg-10Zn-0.8Ca-0.5Zr (wt.%) already developed by the present authors [45,46]. The objective is to investigate whether this high-biocompatible material is suitable for biodegradable temporary custom implants obtained via additive manufacturing. Our previous investigations [45,46,47] were related to the optimisation of process parameters for mechanical alloying, for obtaining the alloy in the powder stage, and for additive manufacturing by L-BPF (laser bed powder fusion), the process of obtaining a sample for a demonstrative implant. Now, the investigations have focused on the main functional properties of the alloy, corresponding to all processing stages, from a powder to demonstrative implant, i.e., structural and mechanical characteristics and corrosion resistance in simulated body fluids. 

## 2. Materials and Methods

### 2.1. Temporary Demonstrative Implant Realization 

Temporary demonstrative implants were realised using the selective laser melting (SLM) technique. Therefore, the first necessity was to obtain the alloy in a powder state using the mechanical alloying (MA) process, which consists of milling the powders of pure chemical elements (purity 99.00%): Mg powder having a shape below 0.1 mm; Zn and Zr powders having a shape below 0.04–0.05 mm; Ca granules. The obtained chemical composition was (in wt.%): Mg-10Zn-0.8Ca-0.5Zr. Different milling times were applied, starting with 2 h and ending with 10 h. 

The planetary mill of high energy (a PM 100 Retsch type) was used to obtain the powder mixture; the used parameters were as follows: the capacity of 500 mL; the frequency of 60 Hz; the diameter of ZrO_2_—balls of 10 mm; the weight ratio powder/ball of 10:1, a commonly used ratio in this case; the milling speed of 300 rpm, usually being between 150 and 350 rpm. The variable parameter was the milling time, with subsequent used values: 2 h, 4 h, 6 h, 8 h, and 10 h. To assure a protective atmosphere, it has been used the argon with an overpressure of 1.5 bar. A 5% n-heptane solution has been introduced to obstruct excessive powder cold welding at the time of milling. The intent was to investigate the powder microstructure evolution, both with homogeneity, in the time mechanical alloying procedure. After the MA procedure, a sieving process was applied to obtain powder sizes below 30 µm.

The achieved powder alloy with the finest particles and as round as possible was selected for the subsequent 3D printing processing using the SLM method for obtaining the temporary demonstrative implant specimen. As a result, the alloy in powder state processed for 10 h of milling time (the maximum applied) was found to be the most suitable for the SLM trial. 

For the SLM procedure, the laser MYSINT 100-3D Selective Laser Fusion (SISMA S.p.A., Vicenza, Italy) represents a particular printer used for metal powder, with absorbed maximum power of 1.50 kW; the used inert gas was Argon. The used processing parameters were as follows: laser power between 0.05 and 0.2 kW; laser speed between 0.6 and 1 m/s; layer height between 0.02 and 0.03 mm; laser energy density between 150 and 550 J/mm^3^.

**Figure 1:**
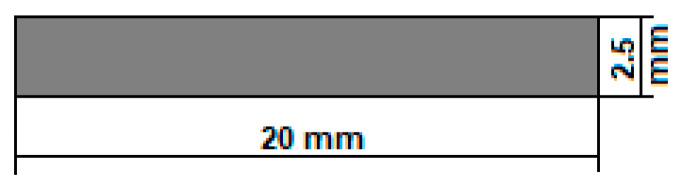
The geometry of the temporary demonstrative implant.

Figure 1 shows the established shape and dimensions of the temporary demonstrative implant. For the geometry, the “pin” type of implant was chosen, a cylinder with 0.25 cm as diameter and 2 cm as length. These dimensions are suitable for both fixations of small human bones and for obtaining fixation screws by precision machining (CAM). They can also be used for in vivo tests on animals, as a spy in the preliminary tests of biocompatibility and hydrogen evolution, or in the medullary canal of fractured bones to follow the healing and the biodegradation process.

The dimensions of the cylindrical samples were calculated based on the following formula, used to calculate the thickness of the implant according to its corrosion rate in simulated body fluids and the time required for the duration of the implant in the human body, correlated with the recovery of the fractured bone [48,49,50]:

(1) $$Thickness\;\left[mm\right]=Corrosion\;Rate\;\left[\frac{mm}{year}\right]\times \frac{Implant\;duration\;[months]}{12\;months\;in\;a\;year}$$

Considering that the corrosion rate is a parameter that is based on various complex factors such as the compositional characteristics of the biomaterial, its density, the exposed area, the immersion time in the simulated body fluids, and their biological characteristics (chlorine, dissolved oxygen and pH level), an average value of approximately 1.5 mm/year was selected, evaluated for our 3D printed and uncoated Mg-based alloy 

In addition, the implant degradation duration of about 20 months was considered (generally, around 1.5–2 years, in function of the implant type) [51]. At least 3 months are necessary to keep strong mechanical support of the damaged bone [51]. It results in a value of 2.5 mm for the cylindrical sample diameter. As for the length of the sample, the established value of 20 mm is also covered and can be used either at this length or adjusted/shortened to a value decided by the surgeon directly during the operation. The temporary demonstrative implant may be customised according to the medical requirements.

### 2.2. The Analysis of the Microstructure and Mechanical Properties of the SLM-Processed Mg-Based Alloy

For the experimented alloy, the analysis of the microstructure (in powder and bulk status) assumed the following steps: (a) the SEM analysis to investigate and calculate the homogeneity and morphology of the achieved samples, in powder state and SLM state as well; A microscope of Tescan VEGA II-XMU SEM type (TESCAN Orsay Holdings, a.s., Czech Republic, Brno-Kohoutovice), with a Bruker QUANTAX xFlash 6/30 EDS detector (USA, Billerica, Massachusetts) was used. The same calibrated microscope was used to measure the dimensions of the powder-alloy particles. (b) The analysis of the X-ray diffraction on the sample in powder state at room temperature was performed using a RIGAKU MiniFlex600 (RIGAKU, Tokyo, Japan) benchtop diffractometer, with Cu-Kα radiation and scattering angle 2θ between 30 and 90° for a step size of 0.02°; the provided detection limit was between 0.1 and 1 wt.%. To perform the analysis, the WPPF (whole powder pattern fitting) was applied, which includes the Rietveld method [52].

The mechanical test of compression was applied on samples after SLM processing using the universal INSTRON 3382 material testing machine (Instron Ltd., High Wycombe, Buckinghamshire, HP123SY, UK). The loading was performed increasingly until the sample failed. The strain–stress curves were determined and analysed. 

### 2.3. Corrosion Analysis of the SLM Processed Mg-Based Alloy

The corrosion behaviour of the SLM-processed Mg-based alloy was analysed using two methods. The first applied was the immersion test for measuring the evolved hydrogen evolution after 4 days of immersion; this test has been carried out in 60 mL Hanks’ Balanced Salt Solution (HBSS, Sigma Aldrich, Merck KGaA, Darmstadt, Germany) at room temperature. The second applied method was the electrochemical corrosion test.

The Autolab Potentiostat/Galvanostat PGSTAT302N (Metrohm, Herisau, Switzerland) and a commonly used cell system with three-electrode were used for the electrochemical corrosion tests. For this system, the testing material was the working electrode, a silver/silver chloride (Ag/AgCl 3M) electrode was employed for the reference electrode, and a platinum one for the counter. Hanks’ Balanced Salt Solution (HBSS, Sigma Aldrich, Merck KGaA, Darmstadt, Germany), pH of 7.0–7.4, was used as electrolyte at room temperature, and Potentio-Dynamic Polarisation tests (PDP) were performed retrieving current-voltage characteristics in a linear sweep mode. The testing parameters were as follows:

- Open Circuit Potential (OCP) stabilisation time of 15 min to ensure stable OCP before PDP tests;
- Polarization range of ±250 mV;
- Scanning rate of 1 mV/s;
- E_corr_ and j_corr_ for the Tafel method of extrapolation;
- Tests performed in triplicate.

## 3. Results and Discussion

### 3.1. Microstructural Investigations and Analysis of the Alloy in Powder State and after SLM Processing 

The results after mechanical alloying are outlined below.

The reason for applying several milling times (2–10 h) consisted of finding the option to obtain a powder with characteristics suitable for the SLM processing as follows: fineness as high as possible; spherical morphologies as possible; without defects, impurities, or porosities; with almost equal diameters. The other important parameters for an efficient MA process, such as the weight ratio of powder to oxide balls or the grinding speed, were optimised in previous research [45,46,47] at 10:1 for the weight ratio and 300 rpm, respectively. Obviously, the variant with a longer milling time (10 h) proved to be necessary to obtain a powder with optimal characteristics. The alloying process during MA and the obtaining of a very fine powder of homogeneous alloy is mediated by the crystallographic defects (point defects, dislocations, stacking faults, etc.) that appeared as a result of cold SPD (severe plastic deformation); all these crystallographic irregularities act as a rapid diffusion medium for obtaining a high solubility of the elementary components. It follows that by choosing the appropriate parameters of the MA process and the chemical composition of the alloy, various alloys can be obtained starting from metal powders [53,54,55].

**Figure 2:**
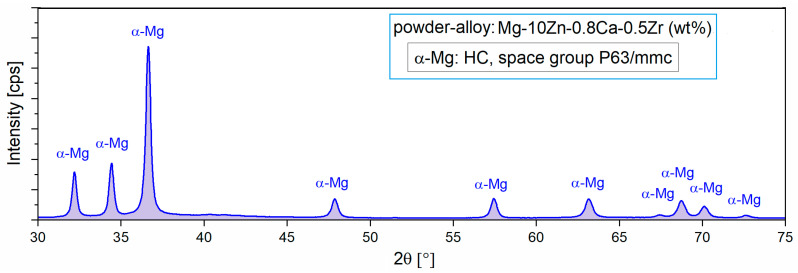
The X-ray diffraction spectra of the studied alloy in powder state obtained via MA for a milling time of 10 h and a milling speed of 300 rpm [47].

For the present case, it appears that by applying the MA process, a homogeneous alloy powder (300 rpm/10 h/10:1) could be obtained, which was then subjected to XRD analysis to reveal the constituent phases. Figure 2 with XRD spectra shows the presence of only the majority α-Mg phase, which means that 10 h of milling time was enough to obtain full solubility for Zn, Ca, and Zr in the Mg matrix.

**Figure 3:**
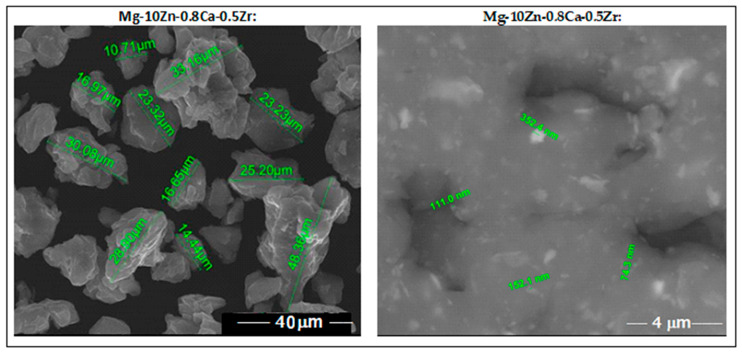
The images obtained via SEM analysis for the studied alloy in powder state obtained via MA for a milling time of 10 h.

SEM analysis, performed after <30 µm sieving of the powder alloy, indicates fine particles with rounded morphology and no impurities or pores. These features observed in Figure 3 represent a good reason for encouraging SLM processing. 

The results after SLM processing of the studied alloy are outlined below.

**Figure 4:**
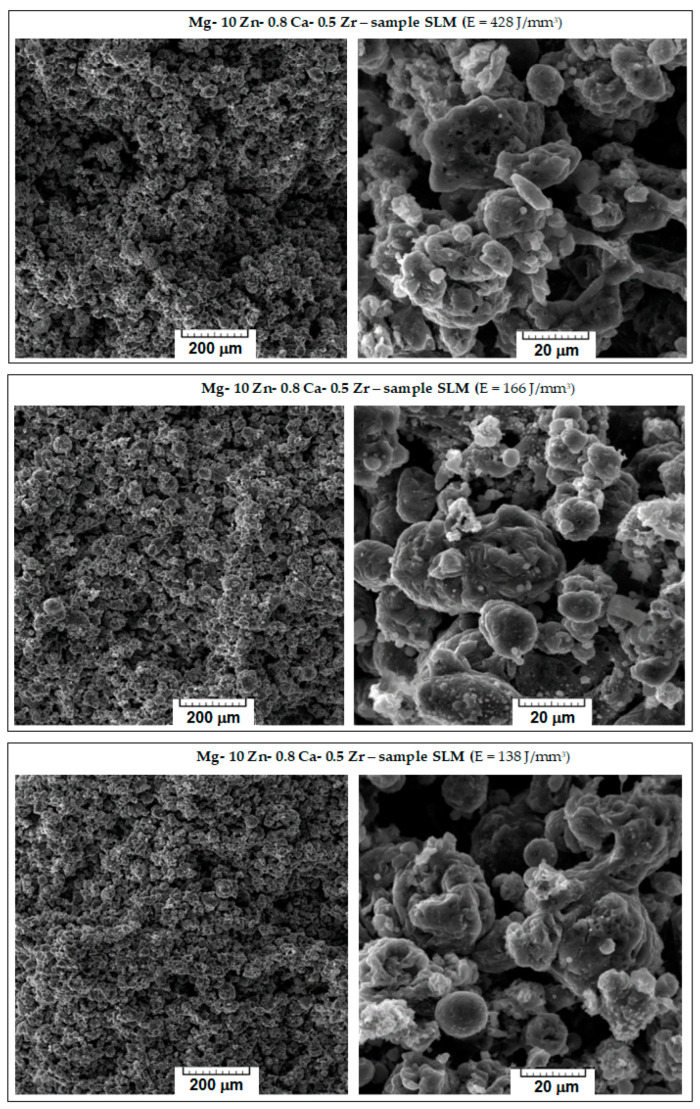
The images obtained via SEM analysis representing the surface of the samples of the studied alloy after processing via SLM method.

The microstructure characteristics of the obtained SLM samples have been analysed using SEM images. From the initial macroscopic examination, the samples with weak compactness, friability, or cracks were removed; Figure 4 shows the microscopic details of the remaining robust samples. They are ordered considering three variants for the decreasing value of the applied laser energy density (E): 428 → 166 → 138 J/mm^3^. With respect to the morphological requirements imposed on temporary biodegradable implants, the goal was to obtain a solid structure but sufficiently porous at the same time, which could imitate the structure of the human bone. Consequently, to appreciate the obtained internal porosity, visual analysis was performed on a breaking surface of the samples. 

Observing Figure 4, it appears that major differences are not seen; all images show a porous morphology. Nevertheless, for the case of high E (top images of Figure 4), the sample was slightly friable with irregular pores compared to samples processed with lower values of E. A significant fact is that the well-known balling effect, which, if occurring, reduces the mechanical performance of SLM-processed samples [56], was not observed for any sample. As a result, the most suitable sample was the one with E = 138 J/mm^3^, on which an additional porosity analysis and a mechanical compression test were performed.

Consequently, an analysis of porosity was performed on a sample with E = 138 J/mm^3^ using the xylene method. For the analysed sample, the initial mass is m_0_ = 0.5248 g. The xylene density is 0.866 g/cm^3^. The sample should be immersed in xylene until saturation. The mass of the sample weighed in the air is M_1_ = 0.7213 g, and the one weighed in xylene is M_2_ = 0.2265 g. Taking these two values into account, a value of 0.6460% was obtained for the apparent porosity (Pap) and 44.19% for the open porosity (Po). When comparing the obtained apparent porosity of 0.64% with the intra-cortical porosity reported to be between 0.028 and 0.06%, depending on gender (men or women) and the type of bone (tibia or radial cortical bone) [57], it can be stated that the result can be considered satisfactory, even if it can be improved by finding a set of SLM parameters to reduce the internal porosity to lower values.

**Figure 5:**
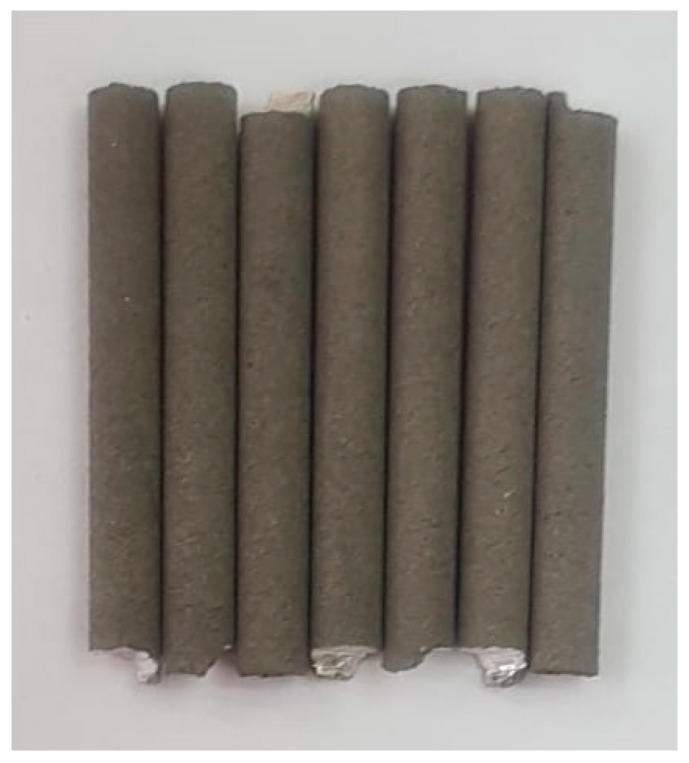
Temporary demonstrative implants realised via SLM from Mg-10Zn-0.8Ca-0.5Zr biodegradable alloy.

Using the SLM parameters with E = 138 J/mm3, several cylindrical samples were made as demonstrative temporary implants, as shown in Figure 5.

### 3.2. Mechanical Properties Analysis of the Alloy in Powder State and after SLM Processing 

**Figure 6:**
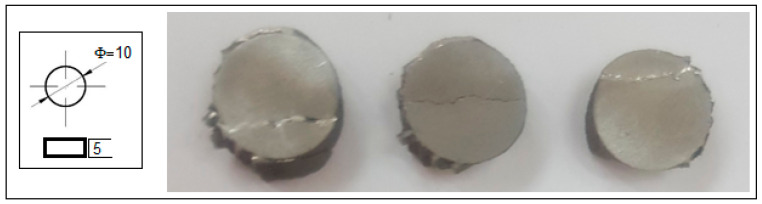
The geometry and the final tested samples to compression of the Mg-10Zn-0.8Ca-0.5Zr biodegradable alloy.

**Figure 7:**
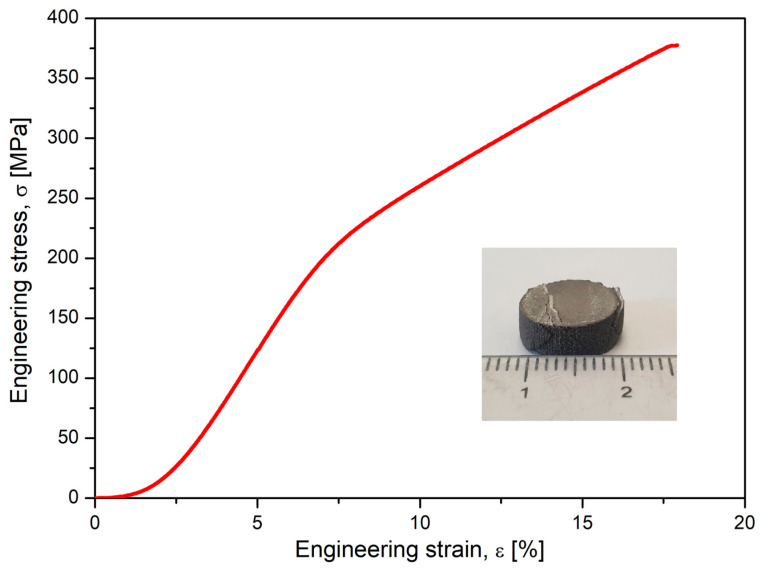
The resulting stress–strain curve after the compressive test performed on studied alloy after SLM processing.

Alongside microstructural analysis, the experimented alloy in the SLM condition with the most solid morphology and minimal pores has been tested for compression. A lot of samples with similar SLM processing and with a geometry shown in Figure 6 were tested, and the results are indicated in Figure 7, which shows the obtained stress–strain curve. The resulting mechanical characteristics are presented in Table 2. As can be seen, the obtained values, compared to those in Table 1, are not similar to those of cortical bone (130–180 MPa) but are much closer than those of other metal alloys used as temporary or permanent implants [1,53,54,55,58].

### 3.3. Corrosion Investigations of the Powder Alloy and SLM Samples 

**Figure 8:**
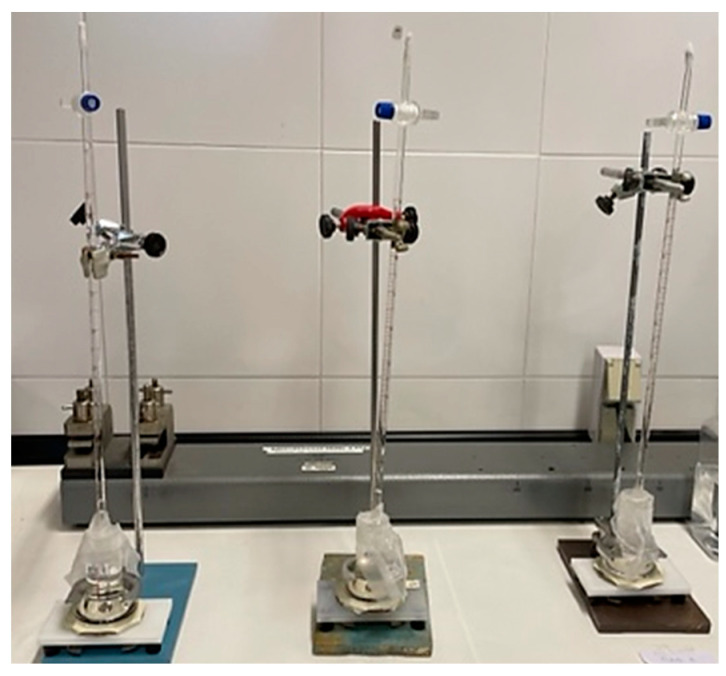
**Figure 8**. Experimental setup used to collect the H_2_ gas produced via the corrosion process.

**Figure 9:**
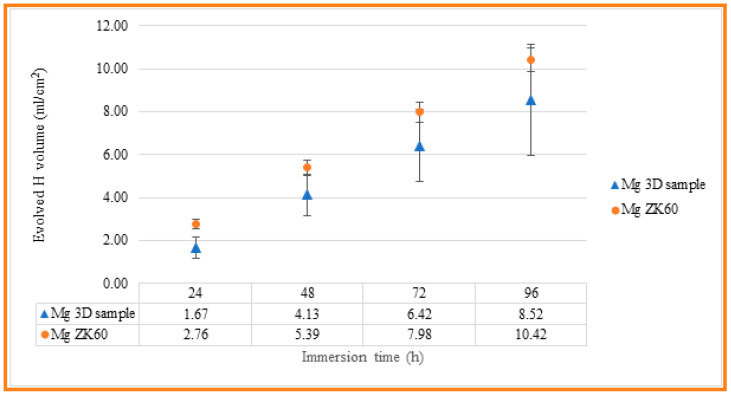
Evolved hydrogen evolution along 4 days of immersion for both 3D-SLM Mg alloy and commercial ZK60 samples.

The corrosion resistance of the uncoated Mg alloy, Mg-10Zn-0.8Ca-0.5Zr (wt.%), has been evaluated in different ways, and the results have been compared with those measured for ZK 60 magnesium alloy, which is typically used in biomedical applications. First, immersion tests have been carried out in 60 mL Hanks’ Balanced Salt Solution (HBSS, Sigma Aldrich) at room temperature in order to measure the evolved hydrogen evolution during 4 days of immersion. Tests were conducted in triplicate, and the samples were masked to leave an exposed surface area of 3 cm^2^ to evaluate the real hydrogen generation captured by an inversed burette. Hydrogen evolution was monitored every 24 h, while the solution was refreshed every 24 h to simulate the renewal of the human body medium [59]. Figure 8 indicates the experimental setup used to collect the H_2_ gas produced via the corrosion process. For the experimental setup, a graduated inverted burette was used, which was placed in a corrosion cell. According to [60,61], it is possible to relate the evolution of 1 mole of hydrogen gas with the dissolution of 1 mole of Mg, so measuring the produced volume of H_2_ gas, mass loss of the Mg can be predicted. The results obtained for the present studied Mg alloy, and for the commercial alloy, ZK 60, are shown in Table 3 and Figure 9, for comparing the corrosion resistance of both materials. 

As can be seen, the evolution of hydrogen for both materials is close, with a smaller volume of hydrogen generated in the case of the SLM-3D sample after 96 hours of immersion, of 8.52 mL/cm^2^, compared to 10.42 mL/cm^2^ measured for the ZK60 magnesium alloy. However, the standard deviation of the results obtained for the SLM-3D samples is higher than that observed for the ZK60 samples, which may be related to their higher surface roughness and, as a result, greater variability of the results. 

**Figure 10:**
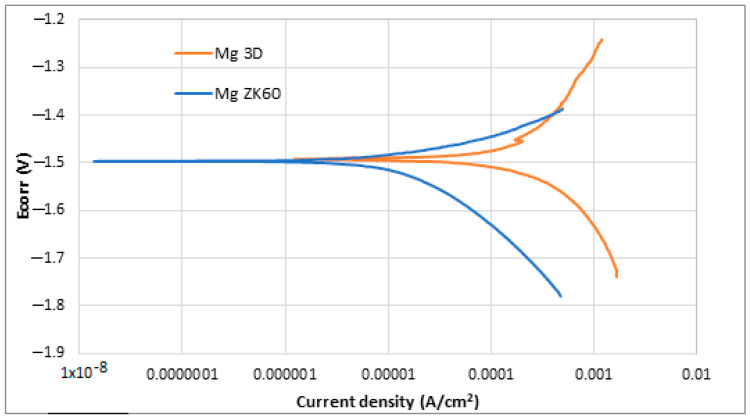
Potentio-dynamic polarisation curves of uncoated ZK60 magnesium alloy (blue) and newly developed 3D Mg samples (orange).

Also, the Potentio-Dynamic Polarisation tests (PDP) were performed. The results obtained after the PDP tests and the application of the Tafel extrapolation method are shown in Table 4, while the PDP plots obtained in the corrosion tests are shown in Figure 10. 

Observing both Table 4 and Figure 10, it is possible to note that both samples presented a similar behaviour against corrosion. The corrosion potential (E_corr_) is almost the same for both alloys, −1.49 and −1.50 V, while current density (j_corr_) is slightly lower in the case of ZK 60 magnesium alloy. More positive corrosion potential values are supposed to be indicative of the retirement for corrosion initiation tendency [62,63]. In general, it is known that for a better corrosion performance of a material, the E_corr_ should be high, and the j_corr_ should be low [64]. In the case of current density, lower values suppose higher corrosion resistance. ZK60 Mg alloy presented the lowest current density (22 ± 5 µA/cm^2^), while the studied SLM-3D Mg alloy presented a value of 68 ± 2 µA/cm^2^. In the last column of Table 4, it has been added also the corrosion rate expressed in [mm/year] in order to have a more complete image of the measurement units for corrosion. It is observed that the corresponding value for the studied alloy is higher than that of the commercial ZK 60 alloy. For the commercial ZK 60 alloy, the corrosion rate, already reported in other experimental works, is similar to that obtained in this present work [65,66,67,68,69,70]. Despite the difference, it can be said that both materials have a similar trend as they are within the same order of magnitude. The higher surface roughness of the samples made via SLM could be the reason for this worse corrosion resistance.

## 4. Conclusions

(a) In order to obtain demonstrative biodegradable temporary implants from magnesium alloy, a new chemical composition was chosen: Mg-10Zn-0.8Ca-0.5Zr (wt.%).
(b) The innovative method of obtaining these implants falls into the category of 3D additive manufacturing, for which the SLM option was chosen, which allows for obtaining customised geometries of the implants. For the present case, a simple geometry was chosen in the form of small “pins”; the length is 20 mm, and the diameter is 2.5 mm.
(c) The alloy powder required for SLM processing was obtained by mechanical alloying, for which the variant with a grinding time of 10 h proved to offer the finest powder with a homogeneous structure and shapes close to round, characteristics highlighted via the following microstructural analyses performed: XRD and SEM.
(d) By varying the laser energy density E [J/mm^3^], which for SLM processing represents a crucial parameter, several types of SLM samples were obtained, but the most robust, consistent/intact and without cracks proved to be the one that used the lowest energy density, of 138 J/mm^3^. One of the processing conclusions indicates that a low laser power is associated with a moderate scanning speed. As a result, the resulting apparent porosity of about 0.64%, measured inside the SLM samples can be considered satisfactory if compared with the intra-cortical porosity reported to be between 0.028 and 0.06%.
(e) The compression test performed on SLM samples highlighted results that fall within the general values of magnesium alloys tested by others and very close to those of cortical bone.
(f) Considering the questionable corrosion resistance of the studied magnesium alloy, future studies and experiments with alloy samples covered with protective layers capable of delaying the corrosion rate are required. Thus, the total degradation time could be enlarged.

## Figures and Tables

**Table 1:** Mechanical characteristics of different metallic implant materials versus natural bone [1].

Characteristic	Cortical Bone	Ti Alloys	Co-Cr Alloys	Stainless Steel	Mg Alloys
Density [g/cm^3^]	1.8–2.1	4.4–4.5	4.47	7.8	1.74–2.0
Elastic modulus [GPa]	3–20	110–117	230	189–205	41–45
Yield strength [GPa]	130–180	758–1117	450–1000	170–310	85–190

**Table 2:** Mechanical characteristics (ultimate tensile strength—*σ_max_*; maximum elongation—*ε_max_*; the elastic modulus—*E*) of the studied Mg alloy after compression test.

	*σ_max_*, [MPa]	*ε_max_*, [%]	*E,* [GPa]
Mg-10Zn-0.8Ca-0.5Zr Alloy in SLM Condition	377.23	17.77	42.60

**Table 3:** The evolved hydrogen evolution along 4 days of immersion for both 3D-SLM Mg alloy and commercial ZK60 samples.

	Evolved Hydrogen Volume (mL/cm^2^)			
	24 h	48 h	72 h	96 h
3D-SLM—Mg alloy	1.67 ± 0.9	4.13 ± 0.96	6.42 ± 1.68	8.52 ± 2.58
ZK 60	2.16 ± 0.24	5.39 ± 0.37	7.98 ± 0.46	10.42 ± 0.53

**Table 4:** Results obtained in the Tafel extrapolation after potentio-dynamic polarisation tests in HBSS for studied SLM-3D-Mg alloy sample and Mg ZK60 alloy.

	Ecorr (V)	j_corr_ (µA/cm²)	Rp (Ω)	Corrosion Rate (mm/year)
3D Mg alloy	−1.49 ± 0.002	68 ± 2	56 ± 11	1.47 ± 0.04
ZK 60	−1.50 ± 0.015	23 ± 5	71 ± 19	0.49 ± 0.10

## Data Availability

The data presented in this study are available upon request from the corresponding author.
